# Supplementary material for: Proactive distractor suppression in early visual cortex
Source: eLife. 2025 Mar 17;13:RP101733. doi: 10.7554/eLife.101733 (PMC11913444; doi:10.7554/eLife.101733)
Supplement: Supplementary file 1. — Contrasted are the three stimulus locations (high-probability distractor location [HPDL], neutral location nearby the HPDL [NL-near], neutral location furthest away from the HPDL [NL-far]) for each stimulus type (distractor, target, neutral stimulus) separately. Reported are paired t-tests or Wilcoxon signed-rank tests results as appropriate with associated effect sizes (Cohen’s d for t-tests and matched rank biserial correlation for Wilcoxon signed-rank tests). p-Values are uncorrected. Bayes factors denote the BF10 from Bayesian paired t-tests. [file elife-101733-supp1.docx]

| **Stimulus type** | **Location contrast** | **Test statistic** | **P value** | **Effect size** | **Bayes factor** |
| --- | --- | --- | --- | --- | --- |
| Distractor | HPDL vs NL-near | *t*_(27)_ = -0.20 | *p* = 0.847 | *d* = -0.04 | BF_10_ = 0.20 |
|  | HPDL vs NL-far | *W* = 94 | *p* = 0.012 | *r* = -0.54 | BF_10_ = 5.99 |
|  | NL-near vs NL-far | *W* = 310 | *p* = 0.014 | *r* = -0.53 | BF_10_ = 4.00 |
| Target | HPDL vs NL-near | *W* = 162 | *p* = 0.362 | *r* = -0.20 | BF_10_ = 0.35 |
|  | HPDL vs NL-far | *W* = 57 | *p* < 0.001 | *r* = -0.72 | BF_10_ = 41.78 |
|  | NL-near vs NL-far | *W* = 327 | *p* = 0.004 | *r* = -0.61 | BF_10_ = 6.27 |
| Neutral | HPDL vs NL-near | *t*_(27)_ = -0.39 | *p* = 0.696 | *d* = -0.08 | BF_10_ = 0.22 |
|  | HPDL vs NL-far | *t*_(27)_ = -2.99 | *p* = 0.006 | *d* = -0.57 | BF_10_ = 7.26 |
|  | NL-near vs NL-far | *W* = 302 | *p* = 0.023 | *r* = -0.49 | BF_10_ = 1.90 |

Supplementary File 1.
